# Accuracy of Four Intra-Oral Scanners in Subgingival Vertical Preparation: An In Vitro 3-Dimensional Comparative Analysis

**DOI:** 10.3390/ma16196553

**Published:** 2023-10-04

**Authors:** Alessio Casucci, Giulia Verniani, Ralph Habib, Nicolò Maria Ricci, Clelia Carboncini, Marco Ferrari

**Affiliations:** 1 Department of Prosthodontics, University of Siena, 53100 Siena, Italy; alessio.casucci@gmail.com (A.C.); giuliaverniani96@gmail.com (G.V.); ralphhabibdr@gmail.com (R.H.); riccinicolomaria@gmail.com (N.M.R.); 2 Department of Periodontics, University of Turin, 10126 Turin, Italy; fabiocarboncini@me.com

**Keywords:** intraoral scanners, subgingival preparation, vertical preparation, accuracy, digital impression

## Abstract

One of the most critical aspects in intraoral impression is the detection of the finish line, particularly in the case of subgingival preparations. The aim of this in vitro study was to evaluate the accuracy among four different Intra Oral Scanners (IOSs) in scanning a subgingival vertical margins preparation (VP). A reference maxillary typodont (MT) was fabricated with a VP for full crown on #16 and #21. The MT was scanned with a laboratory scanner (Aadva lab scanner, GC, Tokyo, Japan) to obtain a digital MT (dMT) in .stl format file. A group of 40 digital casts (dIOC) were obtained by scanning the MT 10 times with four different IOSs: Trios 3, 3Shape A/S; I700, Medit; Vivascan, Ivoclar; and Experimental IOS, GC. All the obtained dIOCs were imported into an inspection software program (Geomagic Control X; 3D SYSTEMS) to be superimposed to the dMT in order to calculate trueness. Therefore, in order to calculate precision, all the scans of the same scanner group were superimposed onto the cast that obtained the best result of trueness. The results were collected as the root mean square value (RMS) on the #16 and #21 abutment surfaces and on a marginal area positioned 1 mm above and below the gingival margin. A nonparametric analysis Kruskal–Wallis test was performed to compare the RMS values obtained in the different iOS groups for trueness and precision. Statistical significance was set at 0.05. For the trueness on the #16 abutment, the Vivascan reported statistically lower values, while on the #21 abutment, Vivascan (56.0 ± 12.1) and Experimental IOS, GC (59.2 ± 2.7) performed statistically better than the others. Regarding precision, Experimental IOS, GC were significantly better than the others on #16 (10.7 ± 2.1) and in the #21 area Experimental, GC, and Trios 3 performed statistically better(16.9 ± 13.8; 18.0 ± 2.7). At the subgingival marginal level for both #16 and #21, all the IOS reported reduced accuracy compared to clinical acceptance.

## 1. Introduction

The use of digital technology in dentistry has increased in recent years [1], thanks to different computer-aided design and computer-aided manufacturing (CAD-CAM) systems being used to fabricate different types of prostheses [2] and to intraoral scanners (IOSs) that allow us to obtain a full digital workflow from the impression to the delivery.

In a completely digital workflow, an accurate IOS is an essential aspect for long term results, since it can guarantee a proper fitting of the future restorations [3].

Recently the IOS’s clinically acceptable results were shown on the fabrication of crowns and fixed partial dentures (FPDs) [3,4,5], with higher time efficiency and better patient acceptance compared with those of conventional impression methods [6,7]. As reported in the glossary of digital terms [8], the accuracy of a digital scanner is the closeness of agreement between a measured result and a reference value. It is described using trueness and precision. Trueness is the closeness between the test object and the reference object, whereas precision is the variability of repeated measurements of the object [9,10]. Differences in accuracy have been reported between IOSs and laboratory scanners [11], and among different IOSs [12,13]. Additionally, the accuracy of an IOS can be affected by clinical circumstances such as the scanning protocol [14], the presence of blood or saliva [15], limited spacing between the abutments and adjacent teeth [16], and edentulous span length [17]. One of the most critical steps during impression taking, both conventional and digital, is detecting the finish line, particularly in subgingival tooth margins. For both the traditional or digital impression techniques, the detection of the finish line relies on a clean and healthy gingival sulcus, proper soft tissue displacement, and clear visibility of the prepared tooth anatomy. However, the preparation of an abutment for a digital impression must consider limitations due to the digital impression device [18]. To date, only few studies [15,19,20,21,22] evaluated the reliability of intraoral scanners in detecting subgingival vertical preparations (VP). So, the aim of this in vitro study was to evaluate the trueness and precision of four IOS devices: Trios 3 (3Shape A/S, Copenhagen, Denmark); I700 (Medit, Seoul, Republic of Korea); Vivascan (Ivoclar Schaan, Liechtenstein); and Experimental IOS (GC, Tokyo, Japan), which are used in standardized conditions on complete crown abutments with a subgingival VP finishing line and with particular attention to the subgingival surface of the preparation. The following null hypotheses were tested: (1) there are no differences in terms of trueness and precision among the different IOSs for the abutment surface, and (2) there are no differences between the tested IOSs in term of accuracy at the subgingival marginal area. 

## 2. Materials and Methods

**Figure 1:**
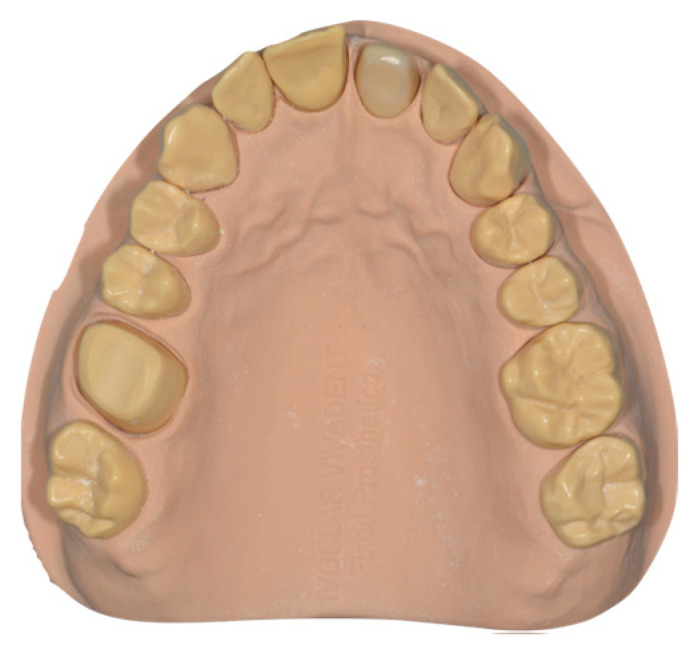
MT model.

A reference maxillary typodont (MT) mounted on a simulator phantom head was fabricated by performing a vertical preparation for full coverage on resin abutments on maxillary right first molar #16 and left first incisor #21. Teeth preparations were performed with the following protocol: mesio-distal preparation with a flame bur 012 (Komet, Lemgo, Germany) preparation of the occlusal surface following the angle of the cusps using a conical burr (Komet, Lemgo, Germany), and axial reduction above the buccal and palatal cemento-enamel junction with the 012C flame diamond burr. Thus, a circumferential tooth reduction was obtained using a flame bur 012C vertically below the cemento-enamel junction until the preparation is rectified with the axial plane. In order to standardize the scanning condition, the preparation was performed at least 2 mm around the gingival margin to ensure the overcome clinical limit, which was confirmed with a periodontal probe (CP 15 UNC; HU-Friedy, CHI, Chapel Hill, NC, USA). The final MT model is shown in Figure 1.

The MT was scanned with a laboratory scanner (Aadva lab scanner, GC, Tokyo, Japan) to obtain a digital maxillary typodont (dMT) in standard tessellation language .stl format. 

**Figure 2:**
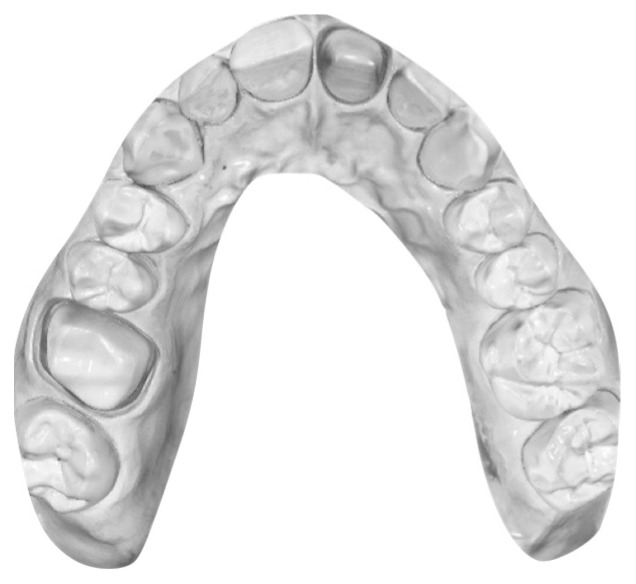
dMT after standardization with Meshmixer.

Subsequently, 40 digital casts (dIOC) were obtained by scanning the MT 10 times using each of the four different IOSs: Trios 3, (3Shape A/S, Copenhagen, Denmark); I700 (Medit, Seoul, Republic of Korea); Vivascan (Ivoclar, Schaan, Liechtenstein); and Experimental IOS (GC, Tokyo, Japan). The scanning procedure was conducted starting from the right maxillary quadrant and ending at the left one, and then continuing on the palatal side and finally on the palatal vault with a clockwise movement. All the scans were performed under the same light conditions and by the same operator with an interval of 10 min to rest and allow the IOS to cool. All the excess areas were removed by using CAD software (Meshmixer; Autodesk, San Rafael, CA, USA) so that the acquired test models were standardized and ready for superimposition, as reported in Figure 2.

The two groups of .stl files dMT and dIOC were imported into an inspection software program (Geomagic Control X, 2018; 3D SYSTEMS) to be superimposed, indicating the dMT “as reference data” in the software program in order to calculate trueness. The dMT .stl file was superimposed with each dIOC .stl file in the software program by activating the function “initial alignment” and then the function “best-fit alignment,” which aligned the two digital casts with a minimal distance between the superimposed surfaces [23]. A 3D analysis was performed on the prepared teeth, #16 and #21 (all regions above the finish line of abutment), and the marginal region (the region up to 1.5 mm on the gingival margin) of the abutment. 

The correspondence between dMT and”dIOC’was evaluated by using the 3D comparison function. The root mean square value (RMS) was calculated based on all cloud points of dMT by using the following formula:

$$RMS=\frac{1}{\sqrt{n}}\times \sqrt{\sum_{i=1}^{n}{\left(x_{1,i}-x_{2,i}\right)}^{2}}$$

where *X*_1,*i*_ indicates a measurement point at *i*th in dMT and *X*_2,*i*_ indicates a measurement point at ith in dIOC. n is the number of all points evaluated. Therefore, the RMS value is the absolute average distance of all cloud points and means of the degree of agreement between dMT and dIOC, so this value was used to evaluate the trueness. 

For each experimental group, the trueness was calculated taking the RMS value resulting from the superimposition of each dIOC .stl and the dMT .stl. The precision was evaluated as the RMS values recorded after the superimposition between each dIOC and the cast that recorded the best result of trueness in the same group. Therefore, all the scans from the same scanner group were superimposed onto this selected cast, whose trueness corresponded to the actual reference value for precision. 

**Figure 3:**
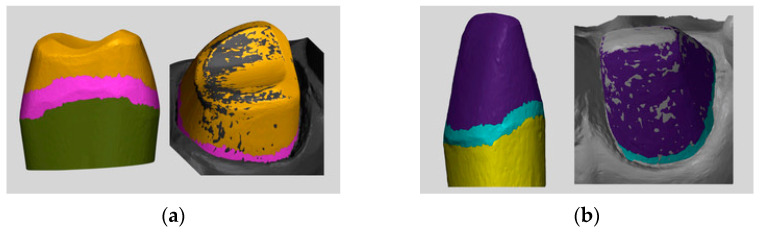
(**a**) Marginal selection area on #16; (**b**) marginal section area con #21.

In order to evaluate the difference in the subgingival marginal area, the single prepared abutments models for 16 and 21 were selected, as reported in Figure 3. The 3D comparison was performed as previously reported after the alignment of the abutment model with the 10 different scans were obtained per each group.

All RMS data were statistically analyzed to evaluate trueness and precision. The homogeneity and normality of distributions were tested via the Kolmogorov–Smirnov method. The nonparametric Kruskal–Wallis test was performed to compare the trueness and precision differences among the scanner groups (α = 0.05). All statistical analyses were performed by using a statistical software program (IBM SPSS Statistics, v26; IBM Corp, Armonk, NY, USA).

## 3. Results

The numbers of images per scan varied between 743 and 1126, and the scanning time was between 1 and 2 min.

### 3.1. RMS Evaluations

The mean RMS values and standard deviations of each group regarding the trueness and precision on the prepared abutments are reported in Table 1.

On the #16 abutment, Experimental IOS, GC performed the best trueness result (55.4 ± 5.6 µm), but no statistically significant differences were found in comparison to the other tested groups except with Vivascan (*p* = 0.003) that performed statistically worse than the others. On the #21 abutment, Vivascan (56.0 ± 12.1 µm) and Experimental IOS, GC (59.2 ± 2.7 µm) performed statistically better than the other two devices for trueness. Regarding precision, Experimental IOS, GC (10.7 ± 2.1 µm) showed statistically better results than the other groups on molar #16, while on incisor abutment #21, the ones that reported statistically better results were Experimental IOS, GC (16.9 ± 13.8 µm) and Trios 3 (29.8 ± 3.7 µm). 

### 3.2. Accuracy at the Subgingival Marginal Level 

The RMS mean values and standard deviations of each scanner at the subgingival marginal level of the prepared abutments are reported in the Table 2.

The I700 reported the highest accuracy at the subgingival marginal level on #16 (96.3 ± 0.13). The I700, Trios 3 and Experimental IOS, GC scanners obtained statistically different and better results than Vivascan at the marginal level for # 21. 

The comparisons between trueness on 16 and 21 and their respective subgingival marginal areas reported a statistical difference in all the IOS groups.

### 3.3. Color Map Evaluations

**Figure 4:**
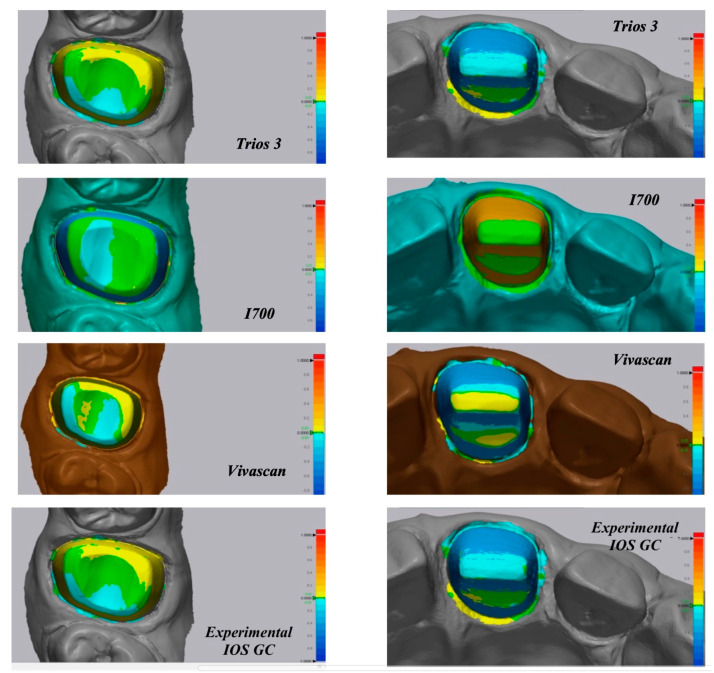
3D trueness analysis of molar and incisor made using different IOSs.

**Figure 5:**
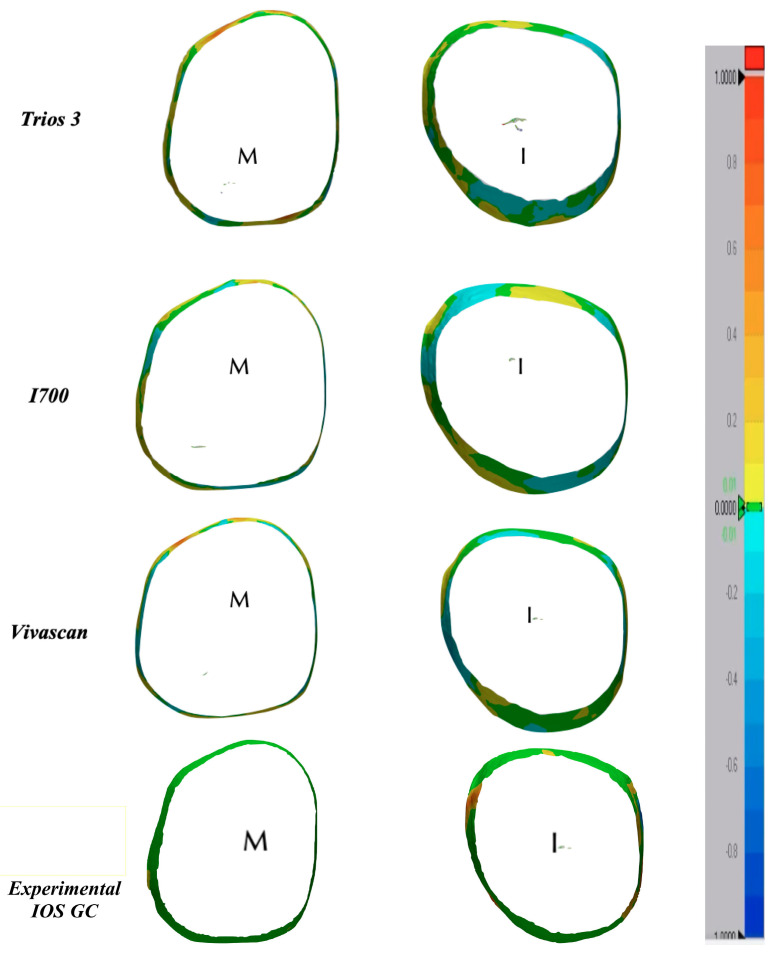
3D analysis on the sub-gingival finish line.

A color map was created to visualize the displacement between the superimposed IOS to MT for the whole abutment area, as shown in Figure 4, and for the sub gingival margin, as shown in Figure 5. The color scale used to highlight the discrepancies is from blue to red and respectively from −100 microns to 100 microns of discrepancy between the two superimposed files. The red-orange areas highlighted the discrepancies between 0.5 µm and 1 mm, while the green areas are the ones perfectly superimposed where the discrepancy is 0 µm. The yellow or light blue areas represent minor discrepancies of ±0.2 µm. 

## 4. Discussion 

In the digital workflow, the accuracy of the cast obtained via IOS becomes fundamental for long-term results [5], in order to achieve a good marginal and internal fit of the restoration [24,25].

The internal fit of the restoration, if incongruous, can lead to precontacts between the restoration’s material and some areas of the abutment, a thick layer of cement along the surface, and ultimately, an exposition of cement at the margin. Marginal fit is one of the main factors in the success of the restoration because any discrepancy leads to a marginal gap and, subsequently, to possible microleakage, cement dissolution by oral fluids, and biofilm accumulation, with consequences such as caries or endodontic and periodontal problems [26]. The maximum width of the marginal gap has not been universally set with precision; many studies consider acceptable clinical gaps until 200 μm, but fixed restorations with marginal discrepancies of less than 120 μm are considered more likely to be successful [27]. It should be considered that in a clinical environment, it would be difficult to translate the μm measured in an in vitro study, and, for this reason, the universally accepted clinical level is difficult to set; it should be as low as possible. 

In a previous in vitro study by Verniani et al. [28], they evaluated the marginal fit of crowns fabricated with a completely digital workflow of vertical preparation. It was reported that the obtained crowns had good adaptation to the abutment independently from the two tested iOS; however, the accuracy at the subgingival finish line was not evaluated. 

To date, only few studies have evaluated the accuracy of IOSs depending on the finishing line location and the difficulties in acquiring subgingival margins, and they compared only a few devices [19,20,21,22].

Due to the lack of evidence in the evaluation of IOS behaviors in vertical preparation, the aim of the present study was to assess the accuracy of different IOSs on the complete abutment surface and on the sub gingival area in vertical prepared abutments. The evaluated IOS devices reported statistically different results for trueness and precision for both #16 and #21; thus, the null hypotheses were rejected. 

Regarding the level of accuracy of complete abutments, all the reported values were largely lower than 100 µm, which was indicated in previous clinical trials studies as the clinically acceptable margins, and, consequently, the recommended scan accuracy [29,30]. The result of this study suggests that, while for the trueness in the molar area, no statistically significant difference was shown between Experimental IOS, GC, Medit I700 or Trios 3 except for Vivascan, which performed statistically worse. When the incisor abutment was evaluated, the Vivascan and the Experimental IOS, GC showed statistically significative and better results compared to the other tested intraoral scanner.

It can be supposed that the proximity of the molar abutment to the adjacent teeth in the posterior area acts as a confounding factor that can modify the performances of the IOSs [16].

On the other hand, in the incisor area, thanks to the increased interproximal space among the abutment and the adjacent teeth, the effect of this confounding factor can be reduced; thus, some statistical differences were found in the RMS-obtained values.

Also, in both #16 and #21 abutments, the interproximal margins were significantly affected in the presence of adjacent teeth and a lower accuracy resulted in respect to the vestibular and palatal marginal sites. This is still referable to the limited space between the scanned surface and the adjacent tooth, as described by Keeling et al. [16].

Regarding precision, Experimental IOS, GC reported statistically lower results than the others IOS devices in both the molar and incisor abutments, revealing the closure scans in between each of the same group, thus resulting as the most repeatable and reliable IOS. Instead, Vivascan reported the biggest standard deviations or precision.

All our data about the sub gingival marginal region reported increased values of trueness and precision compared to the full abutment. The mean values for trueness and precision are all above the level of clinical acceptability according to Shim et al. [30], except for i700 on marginal M.

As it can be evaluated in the color map images in Figure 4 and Figure 5, the prevalence of cold colors at the marginal level revealed as the IOS abutment surface did not penetrate into the reference scan surface. Thus, it seems that the IOS was not able to record the true abutment surface into the sulcus when, at the marginal level, it was closer to the tissue surface. A possible explanation of this finding is related to the continuous surface generated in the software by “joining the dots” according to the “stitching” algorithm.

This behavior of IOS was confirmed by recent papers. Son et al. [21] reported that the trueness of the subgingival marginal region at the location of the subgingival finish line (0.5-mm below the level of the gingival) was the worst. In another study [20], the two IOSs tested showed clinically acceptable scan trueness at a depth of up to 0.25 mm of the subgingival finish line without the gingival displacement cord, but also showed clinically acceptable scan trueness at a depth of up to 1 mm when the gingival displacement cord was used. Additionally, they found out that with the increase in the subgingival finish line depth without the gingival displacement cord, the surface area of the abutment decreased, but they limited the study to only two different types of intraoral scanners. 

Our data also confirm the results obtained by Ferrari Cagidiaco et al. [19] that digital impression is not recommended when the crowns’ margins are positioned deep (1.5–2 mm) into the sulcus.

However, further studies could be conducted in order to understand what the vertical limit for each IOS is to obtain an acceptable scan in terms of accuracy at the marginal level.

Additionally, the present study has some limitations, like the absence of saliva, blood, and limited mouth opening and movements of the patient [31]; those factors could be considered in an in vivo experimental design. 

Also, it must consider that three devices were using a software already available, while the Experimental IOS, GC was an experimental software not available in the market yet.

## 5. Conclusions

Based on the findings of this in vitro study, the following conclusions can be drawn:

1. The trueness deviations of the analyzed scanners were significantly different in the full abutment surface of the molar and incisor.
2. At the subgingival marginal level, the accuracy results were not clinically acceptable for all the IOS, probably due to the “joining the dots” effect.
3. More studies are required to validate the behavior of IOS in vertical preparations.

## Figures and Tables

**Table 1:** RMS mean values and standard deviations of each scanner obtained for trueness and precision in 16 and 21 abutments.

	Trueness #16 [µm]	Trueness #21 [µm]	Precision #16 [µm]	Precision #21 [µm]
Trios 3	60.2 ± 4.9 ^a^	68.7 ± 4.0 ^b^	31.7± 13.1 ^bc^	18.0 ± 2.7 ^ab^
I700	58.0 ± 8.9 ^a^	83.3± 5.6 ^c^	15.8 ± 2.7 ^b^	29.8 ± 3.7 ^b^
Vivascan	69.6 ± 6.9 ^b^	56.0 ± 1.21 ^a^	41.4 ± 20.2 ^c^	49.9 ± 19.6 ^c^
Experimental IOS, GC	55.4 ± 5.6 ^a^	59.2 ± 2.7 ^a^	10.7 ± 2.1 ^a^	16.9 ± 1.3 ^a^

Statistical significative values are reported with different letters a, b or c (*p* < 0.05).

**Table 2:** Mean value and standard deviation of the accuracy of the different IOSs at the marginal level.

	Marginal #16 [µm]	Marginal #21 [µm]
Trios 3	166.0 ± 0.34 ^b^	147.4 ± 2.18 ^a^
I700	96.3 ± 0.13 ^a^	154.2 ± 1.89 ^a^
Vivascan	141.2 ± 2.20 ^b^	170.0 ± 1.33 ^b^
Experimental IOS, GC	145.2 ± 1.87 ^b^	135.7 ± 0.825 ^a^

Statistical significative values are reported with different letters a or b (*p* < 0.05).

## Data Availability

The data presented in this study are available on request from the corresponding author. The data are not publicly available due to University policy.
